# Hydroxymethylated cytosine in development and disease

**DOI:** 10.1186/1756-8935-6-S1-O8

**Published:** 2013-03-18

**Authors:** Gerd P Pfeifer

**Affiliations:** 1 Beckman Research Institute of the City of Hope, Duarte, CA 91010, USA

## 

5-methylcytosine (5mC) is oxidized to 5-hydroxymethylcytosine (5hmC) by Tet proteins. The paternal 5mC is converted in fertilized oocytes to 5hmC allowing for epigenetic reprogramming of sperm DNA. Human tumors contain much lower levels of 5hmC compared to normal tissue. Proliferating cells in a tumor have drastically reduced levels of 5hmC, suggesting that this stable base modification is lost during DNA replication. 5hmC is most abundant in cells of the nervous system. We investigated patterns of 5mC and 5hmC during neurogenesis in the embryonic mouse brain. 5hmC levels increase during neuronal differentiation and this modification associates preferentially with gene bodies of activated neuronal function-related genes, in which gain of 5hmC is often accompanied by loss of H3K27me3. Importantly, gain of 5hmC is rarely associated with DNA demethylation suggesting that 5hmC is a rather stable epigenetic mark. Functional perturbation of the H3K27 methyltransferase Ezh2 or of Tet proteins leads to defects in neuronal differentiation suggesting that formation of 5hmC and loss of H3K27me3 cooperate to promote brain development. We propose that the function of 5hmC in promoter regions is to “repair” inappropriate de novo DNA methylation but its exact mechanistic role in gene bodies is still unknown.

